# Protists Within Corals: The Hidden Diversity

**DOI:** 10.3389/fmicb.2018.02043

**Published:** 2018-08-31

**Authors:** Camille Clerissi, Sébastien Brunet, Jeremie Vidal-Dupiol, Mehdi Adjeroud, Pierre Lepage, Laure Guillou, Jean-Michel Escoubas, Eve Toulza

**Affiliations:** ^1^Univ. Perpignan Via Domitia, IHPE UMR 5244, CNRS, IFREMER, Univ. Montpellier, Perpignan, France; ^2^McGill University and Génome Québec Innovation Centre, Montréal, QC, Canada; ^3^IFREMER, IHPE UMR 5244, Univ. Perpignan Via Domitia, CNRS, Univ. Montpellier, Montpellier, France; ^4^Institut de Recherche pour le Développement, UMR 9220 ENTROPIE & Laboratoire d’Excellence CORAIL, Université de Perpignan, Perpignan, France; ^5^CNRS, UMR 7144, Sorbonne Universités, Université Pierre et Marie Curie – Paris 6, Station Biologique de Roscoff, Roscoff, France; ^6^CNRS, IHPE UMR 5244, Univ. Perpignan Via Domitia, IFREMER, Univ. Montpellier, Montpellier, France

**Keywords:** holobiont, protists, symbiosis, metabarcoding, blocking primer, Scleractinia, *Pocillopora damicornis*

## Abstract

Previous observations suggested that microbial communities contribute to coral health and the ecological resilience of coral reefs. However, most studies of coral microbiology focused on prokaryotes and the endosymbiotic algae *Symbiodinium*. In contrast, knowledge concerning diversity of other protists is still lacking, possibly due to methodological constraints. As most eukaryotic DNA in coral samples was derived from hosts, protist diversity was missed in metagenome analyses. To tackle this issue, we designed blocking primers for Scleractinia sequences amplified with two primer sets that targeted variable loops of the 18S rRNA gene (18SV1V2 and 18SV4). These blocking primers were used on environmental colonies of *Pocillopora damicornis sensu lato* from two regions with contrasting thermal regimes (Djibouti and New Caledonia). In addition to *Symbiodinium* clades A/C/D, *Licnophora* and unidentified coccidia genera were found in many samples. In particular, coccidian sequences formed a robust monophyletic clade with other protists identified in *Agaricia*, *Favia*, *Montastraea*, *Mycetophyllia*, *Porites*, and *Siderastrea* coral colonies. Moreover, *Licnophora* and coccidians had different distributions between the two geographic regions. A similar pattern was observed between *Symbiodinium* clades C and A/D. Although we were unable to identify factors responsible for this pattern, nor were we able to confirm that these taxa were closely associated with corals, we believe that these primer sets and the associated blocking primers offer new possibilities to describe the hidden diversity of protists within different coral species.

## Introduction

Scleractinian corals build reefs all around the world. The ecological success of corals in the oligotrophic seawater of coral reefs mostly relies on the symbiosis with dinoflagellates (genus *Symbiodinium*). In particular, the symbiosis between corals and *Symbiodinium* takes place within coral cells, where the algal symbionts provide organic compounds to corals through their photosynthetic activity, and in turn receive nutrients and metabolic compounds from their host. *Symbiodinium* is a diverse genus divided into nine clades (Coffroth and Santos, 2005; Pochon et al., 2006; Quigley et al., 2014; Thornhill et al., 2017). Among these clades, five have been identified in coral cells (clades A, B, C, D, and F).

Corals are also associated with a high diversity of microorganisms (bacteria, archaea, fungi, endolithic algae, protozoa, and viruses) (Rohwer et al., 2002; Wegley et al., 2004; Thurber et al., 2017), and the complex formed by coral and the associated microorganisms corresponds to a single entity called the holobiont (Rohwer et al., 2002; Theis et al., 2016). Among them, the bacterial genus *Endozoicomonas* was found in high abundances within many coral species (Bayer et al., 2013; Neave et al., 2016, 2017). This genus was thought to be a beneficial symbiont to corals. Moreover, rare bacterial taxa (genera *Ralstonia* and *Propionibacterium*) were ubiquitous as well (Ainsworth et al., 2015). In addition to *Symbiodinium*, other unicellular eukaryotes (protists) were found to live with corals, including many Stramenopiles (Kramarsky-Winter et al., 2006; Harel et al., 2008; Siboni et al., 2010), several apicomplexans such as *Chromera* and coccidians (Toller et al., 2002; Moore et al., 2008; Janouškovec et al., 2012; Kirk et al., 2013; Mohamed et al., 2018), different fungi (Amend et al., 2012), and different boring microflora (*e.g.*, *Ostreobium*, *Phaeophila*, and *Porphyra*) (Tribollet, 2008b; Pica et al., 2016).

Unlike for *Symbiodinium*, the role of these microorganisms remains unknown within the holobiont. First, they might provide protection against pathogens through the secretion of antimicrobial compounds (Ritchie, 2006; Shnit-Orland and Kushmaro, 2009). Secondly, in addition to *Symbiodinium*, they might also provide metabolic compounds to corals (Kramarsky-Winter et al., 2006; Harel et al., 2008; Siboni et al., 2010). Thirdly, microbial communities might play an important role for coral heat tolerance (Ziegler et al., 2017), and for the ecological resilience of coral reefs (McDevitt-Irwin et al., 2017). As a consequence, these observations suggested that microbial communities contribute to coral health and homeostasis, through the presence of Beneficial Microorganisms for Corals (BMC) (Peixoto et al., 2017).

To date, however, most studies have focused on *Symbiodinium* and coral-associated bacteria. In particular, very little is known concerning the diversity and the role of other protists (Ainsworth et al., 2017), though several studies have shown that they play an important role in the structure and function of marine ecosystems (Thingstad et al., 2008; de Vargas et al., 2015). Previous analyses of protists mainly used non-destructive sampling techniques (microscope, culture) or low-throughput methods for environmental DNA (qPCR, cloning), though these methods were less effective at detecting diversity when compared to mass sequencing of the 18S rRNA gene for example. Because most DNA in coral samples was extracted from the host and the 18S rRNA gene is shared between corals and protists, to date high-throughput studies of protist diversity have been a challenge (Šlapeta and Linares, 2013).

To tackle this issue, we designed blocking primers for Scleractinia sequences in order to decrease their proportions relative to protist sequences. Such an approach was already effective in the study of fish and krill gut contents (Vestheim and Jarman, 2008; Leray et al., 2013), and in the removal of metazoa sequences from seawater community samples (Tan and Liu, 2018). To the best of our knowledge, this is the first time that this strategy has been used on coral samples. These blocking primers targeted regions similar to the reverse primer for each of the two primer sets used to amplify variable loops of the 18S rRNA gene (V1V2 and V4) (Wuyts et al., 2000, 2002; Stoeck et al., 2010).

**FIGURE 1:**
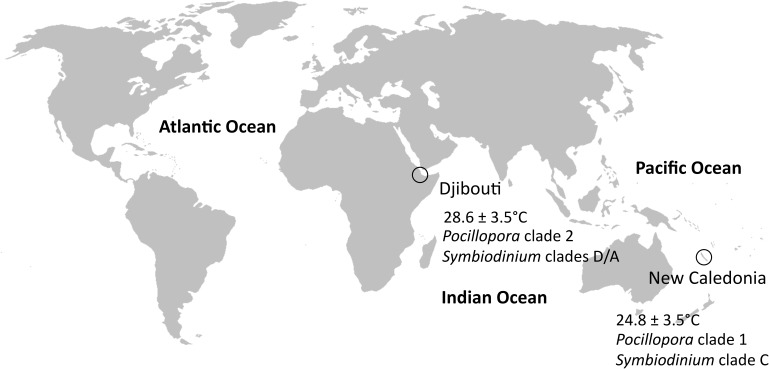
Sampling sites. Djibouti and New Caledonia had different thermal regimes and clades of *P. damicornis* and *Symbiodinium*.

Both blocking primers were used to explore protist diversity within colonies of *P. damicornis sensu lato* that were sampled from two geographic regions with contrasting thermal regimes: Djibouti and New Caledonia (**Figure 1** and **Supplementary Table 1**). A previous study on these samples showed different *Symbiodinium* clades between these regions using ITS2 (internal transcribed spacer 2) (Brener-Raffalli et al., 2018). These authors also highlighted that colonies from Djibouti and New Caledonia corresponded to two different clades of *P. damicornis*. As a consequence, geography, genetics and environmental conditions divided the two *P. damicornis* populations, and allowed for the comparison of different holobionts.

## Materials and Methods

### Sampling Sites

Colonies of *P. damicornis sensu lato* growing between one to five meters depth were sampled by snorkeling within two regions (Djibouti and New Caledonia) in six localities (**Figure 1** and **Supplementary Table 1**). A total of 16 colonies were sampled during this survey. The tip (1–2 cm) from one healthy branch of each colony was cut and placed individually in a plastic bag. Each bag was filled with seawater surrounding the colony to hold samples during the sampling cruise. Samples were subsequently transferred into modified CHAOS buffer (4 M guanidium thiocyanate, 0.5% N-lauryl sarcosine sodium 25 mM Tris–HCl pH 8, 0.1 M β-Mercaptoethanol) as previously described (Adjeroud et al., 2014).

### Design of Blocking Primers for Scleractinia

A preliminary sequencing test was performed to study eukaryote diversity within a sample of *P. damicornis* using two primer sets targeting two differents regions of the 18S rRNA gene, 18SV1V2 and 18SV4 (**Table 1**). While primers for 18SV4 were designed previously to amplify all eukaryotic-specific 18S rDNA (Stoeck et al., 2010), primers for 18SV1V2 were designed using the Protist Ribosomal Reference database (PR^2^) (Guillou et al., 2012) in order to prevent amplification of metazoan 18S rRNA genes especially from *Crassostrea gigas* oysters. Both sequencing tests showed an excess of amplicons from *P. damicornis*, since they represented ∼99% of sequences (for a total of 3383 and 2460 cleaned sequences using 18SV1V2 and 18SV4, respectively; data not shown).

**Table 1:** Primers and blocking primers used in this study.

Marker region	18SV1V2	18SV4
Forward (5′->3′)	ACCTGGTTGATCCTGCCA	CCAGCASCYGCGGTAATTCC
Reverse (5′->3′)	GTARKCCWMTAYMYTACC	ACTTTCGTTCTTGATYRA
Blocking primer (5′->3′)	CTACCTTACCATCGACAGTTGATAG	TCTTGATTAATGAAAACATTCTTGGC
Expected amplicon size (bp)	∼340	∼430

Thus we designed blocking primers for both primer sets in order to reduce the proportion of *P. damicornis* amplicons. First, we downloaded the non-redundant (99%) Silva SSU database (release 128, September 2016) (Quast et al., 2013; Yilmaz et al., 2014). Then we only kept sequences that matched with either the primer set for 18SV1V2 or 18SV4. Based on annotations, metazoa were removed to produce a metazoa-free database and sequences of Scleractinia were used to create a host database. In order to design blocking primers that overlap the reverse primer and the 3′-region of Scleractinia amplicons, we aligned the last 40 nucleotides (corresponding to the 3′-region of amplicon and the reverse primer) of Scleractinia with metazoa-free database using MUSCLE v3.8.31 (Edgar, 2004). Then, we analyzed the nucleotide polymorphism at each position of the alignment for Scleractinia and metazoa-free sequences using entropy decomposition (R package {otu2ot}, CalcEntropy.seq) (Ramette and Buttigieg, 2014). According to previous studies and entropy values (**Supplementary Figure 1**), we designed blocking primers <30 bp with 10 bp overlapping the reverse primer, and having a Tm similar to the targeted primer set (Vestheim and Jarman, 2008; Leray et al., 2013). The best candidate for each primer set (targeting either 18SV1V2 or 18SV4 region) was identified using specificity tests against the Scleractinia and metazoa-free databases. Lastly, these primers were synthesized and modified at the 3′-end with a Spacer C3 CPG (3 hydrocarbons).

### DNA Extraction, PCR, and Sequencing

DNA extractions were performed using CTAB (Cetyl TrimethylAmmonium Bromide)-based extraction method (Winnepenninckx et al., 1993). Briefly, coral tips were lysed 2 h in 600 μL CTAB buffer (2% CTAB, 0.2% β-Mercaptoethanol, 1.4 M NaCI, 20 mM EDTA pH 8, 100 mM Tris–HCl pH 8, 100 μg/mL proteinase K) at 60°C. Thus protists of the whole coral tissues were analyzed in this study. An equal volume of chloroform:isoamyl-alcohol (24:1) was then added. After centrifugation for 10 min at 14,000 *g*, the aqueous phase was transferred to a new tube and DNA was precipitated by adding 400 μL of ice-cold isopropanol and incubating 1 h at −20°C. After centrifugation for 15 min at 14,000 *g*, the supernatant was discarded and the pellet washed with 70% ethanol. The pellet was air-dried for 5 min and resuspended in water. DNA concentration and quality were checked with Epoch microplate spectrophotometer (BioTek Instruments, Inc.).

Then, the 18S rRNA gene of eukaryotic communities was amplified and sequenced using the variable V1V2 and V4 loops (**Table 1**; Wuyts et al., 2002; Stoeck et al., 2010). PCR reactions were carried in a 25 μl volume with final concentrations of 0.4 μM of each PCR primers, 0.02 U of the Qiagen HotStar Taq DNA Polymerase, 0.2 mM of the dNTP mix and 1×Taq buffer. In order to reduce amplification of *P. damicornis* amplicons, blocking primers were added to the PCR mix at a final concentration of 1.2 μM (**Table 1**). PCR cycling included an initial incubation of 15 min at 96°C followed by 35 cycles of 96°C for 30 s, 52°C for 30 s, and 72°C for 1 min, with a final 10 min incubation at 72°C. Paired-end sequencing (250 bp read length) was performed at the McGill University (Génome Québec Innovation Centre, Montréal, QC, Canada) on the MiSeq system (Illumina) using the v2 chemistry according to the manufacturer’s protocol. Raw sequence data are available in the Sequence Read Archive repository under accession ID PRJNA393088 (to be released upon publication).

### Sequence Analyses

The FROGS pipeline (Find Rapidly OTU with Galaxy Solution) implemented into a galaxy instance was used to define Operational Taxonomic Units (OTU), and compute taxonomic annotations (Escudié et al., 2017). Briefly, paired reads were merged using FLASH (Magoc and Salzberg, 2011). After denoising and primer/adapters removal with cutadapt (Martin, 2011), *de novo* clustering was done using SWARM that uses a local clustering threshold, with aggregation distance *d* = 3 (Mahé et al., 2015). Chimera was removed using VSEARCH (Rognes et al., 2016). We filtered the dataset for singletons and performed affiliation using Blast+ against the Protist Ribosomal Reference database (PR^2^) (Guillou et al., 2012) to produce an OTU and affiliation table in standard BIOM format. Because we were interested in studying low frequency OTUs, we used additional steps to remove most PCR and sequencing errors. First, we removed OTUs having an annotation with a blast coverage <90%. Secondly, we computed a phylogenetic tree of the whole OTUs using MAFFT (Katoh et al., 2002) and FastTree (GTR model) (Price et al., 2010). OTUs were removed from the dataset if they corresponded to very long branches on the phylogenetic tree (according to minimal branch length values). Lastly, OTUs were considered present in each sample if they had at least three sequences.

Rarefaction curves of protist species richness were produced using the {phyloseq} R package, and the rarefy_even_depth and ggrare functions (McMurdie and Holmes, 2013). We also used phyloseq to obtain abundances at the genus taxonomic rank (tax_glom function). Pielou’s measure of evenness was computed using affiliated genera and the {vegan} R package.

### Annotation of *Symbiodinium* OTUs

In order to characterize the environmental diversity of *Symbiodinium*, reference sequences of *Symbiodinium* (clades A, B, C, D, and G) were obtained for each 18S rRNA regions from the National Center for Biotechnology Information (NCBI) (**Supplementary Table 2**). In addition, we selected two outgroups: *Polarella glacialis* and *Pelagodinium beii*. The reference were aligned with the environmental sequences of *Symbiodinium* using MUSCLE v3.8.31 (Edgar, 2004), alignments were trimmed at each extremity, and sequences were clustered at different nucleotide similarities (from 90 to 99%) using Mothur (Schloss et al., 2009). The environmental sequences were annotated for a similarity of 95 and 97% for 18SV1V2 and 18SV4, respectively, *i.e.*, when all the reference sequences of *Symbiodinium* clade A, B, C, D, and G clustered into five different groups.

### Alignment and Phylogenetic Analyses

In one hand, phylogenetic reconstructions of *Symbiodinium* reference isolates were carried out to compare phylogenetic signals of both 18SV1V2 and 18SV4 markers with ITS2. We selected two reference sequences of *Symbiodinium* clade A, three reference sequences of clade B, two reference sequences of clade C, one reference sequences of clade D and clade G (**Supplementary Table 3**). Except the strain of clade G, sequences of the three markers were available for each reference isolate of *Symbiodinium*. For each marker, sequences were aligned using MUSCLE v3.8.31 (Edgar, 2004).

On the other hand, phylogenetic reconstructions were computed to describe either environmental *Symbiodinium* diversity, the whole protist genera or coccidians associated with *P. damicornis* samples. First, *Symbiodinium* diversity was studied according to the annotation step of *Symbiodinium* OTUs. In particular, we used the output alignment of Mothur obtained at 95 and 97% similarity cutoffs for 18SV1V2 and 18SV4, respectively. These alignments contained representative OTUs of environmental *Symbiodinium*, the two outgroups (*P. glacialis* and *P. beii*), and the reference sequences of *Symbiodinium* clades. Secondly, sequences of protist genera, *Symbiodinium* clades, and *P. damicornis* (accession number: LT631138.1 for both 18SV1V2 and 18SV4) were aligned using MUSCLE v3.8.31 (Edgar, 2004). Thirdly, we described the diversity of coral-associated coccidians using reference sequences of Apicomplexa (Schrével et al., 2016), and best BLASTn hits of coccidian sequences of this study against the National Center for Biotechnology Information (NCBI).

Finally, all alignments were trimmed at each extremity and maximum likelihood (ML) trees were computed with IQ-TREE v1.3.8 using the best model (selected with the Bayesian information criterion) (Nguyen et al., 2015), and validated via a ultrafast bootstrap procedure with 1000 replicates (Minh et al., 2013).

### Statistical Analyses

All statistical analyses were done using R v3.3.1 (R Core Team, 2008).

First, phylogenetic signals were compared between ITS2, 18SV1V2 and 18SV4 using *Symbiodinium* reference isolates. Patristic distances were obtained from phylogenetic trees (R package {stats}, cophenetic), and compared using Mantel test (R package {vegan}, mantel) (Mantel, 1967).

Secondly, Fisher’s exact tests (R package {stats}, fisher.test) were computed to estimate the association between protist genera and *Symbiodinium* clades with both geographic regions, *i.e.*, Djibouti and New Caledonia.

*P*-values were adjusted for multiple comparisons using the Bonferroni correction ({stats}, p.adjust). The threshold of significance level was set at 0.05.

## Results

### Specificity of Blocking Primers

In order to describe protist diversity associated with *P. damicornis* colonies from Djibouti and New Caledonia, we performed a preliminary sequencing test using one sample and two primer sets targeting different variable loops of the 18S rRNA gene (V1V2 and V4). Since most sequences corresponded to *P. damicornis*, we designed blocking primers using the Silva SSU database (see Methods for more details). These blocking primers targeted 10 bp of reverse primers and ∼15 bp of the amplicon 3′-end of Scleractinia (**Table 1** and **Supplementary Figure 1**). Because their 3′-end had a spacer C3 CPG, elongation is expected to abort, whereas annealing properties are not modified (Vestheim and Jarman, 2008). To estimate the specificity of these blocking primers, we identified sequences of the Silva database that matched with primer sets and blocking primers. We found a very high *in silico* specificity, since 100 and 93.8% of Scleractinia were removed by the 18SV1V2 and 18SV4 blocking primers, respectively (**Table 2**). In addition, they removed very low fractions of protists found in the Silva database.

**Table 2:** *In silico* specificity of blocking primers.

Removed taxa	18SV1V2 (%)	18SV4 (%)
Scleractinia	100	93.8
Rhizaria	1.6	0
Nucletmycea	0.4	<0.1
Alveolata	0.1	0.6
Cryptomonadales	0	0.5
Chloroplastida	0	0.3

**FIGURE 2:**
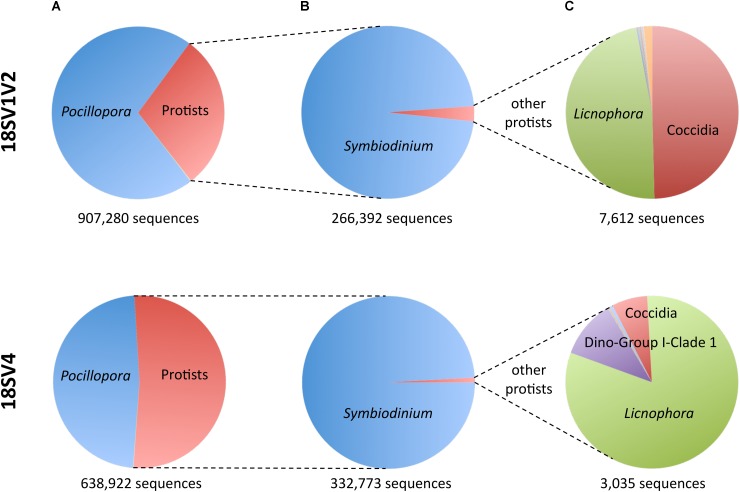
Sequences of *Pocillopora*, *Symbiodinium* and the other protists for both marker regions. **(A)** Fraction of *Pocillopora* compared to protists. **(B)** Fraction of *Symbiodinium* compared to other protists. **(C)** Fraction of the other protists.

As a consequence, we used both primer sets and their blocking primers to study protists associated with the colonies of *P. damicornis* from Djibouti and New Caledonia. Because DNA extraction was done directly on coral tips, we expected to be able to describe the entire protist community (*i.e.*, microbes that were part of the holobiont, and possibly environmental microbes). On average, each sample had ∼29,566 *Pocillopora* sequences and ∼18,724 protist sequences representing ∼26 OTUs (**Table 3** and **Supplementary Table 4**). While *in silico* analyses showed very high specificity of both blocking primers, PCR and MiSeq sequencing displayed higher host contamination (**Figure 2A**). Indeed, *Pocillopora* sequences still represented 71 and 48% of amplicons for 18SV1V2 and 18SV4, respectively. In particular, *Pocillopora* proportion was lower for 18SV4 than for 18SV1V2.

**Table 3:** Number of sequences and OTUs.

Sample	Total number of raw sequences	Total number of cleaned sequences	Total number of OTUs	Number of *Pocillopora* sequences	Number of protist sequences
DJMI5	53724/42120	44953/31038	24/20	29269/11462	15465/19576
DJMI6	90752/30981	74660/16993	29/14	30128/1213	44513/15780
DJMI7	63586/11187	51314/6835	26/11	15387/704	35609/6124
DJRK1	46195/20867	37723/12100	23/11	24296/2204	13408/9896
DJRK3	62498/34901	52163/24230	19/13	38052/8707	14111/15523
DJRK4	63927/51803	54167/40238	18/34	35607/7719	18556/32519
DJRK5	95369/24355	76347/14707	34/17	42900/2156	33419/12551
DJRK7	41294/59287	35298/42366	17/47	25045/6777	10238/35589
DJSB6	102976/68110	88376/48495	25/51	79796/26472	8568/22023
NCBM1	63399/63417	53813/47370	13/45	46548/30929	7256/16340
NCBM3	64930/72320	55578/55268	14/29	44263/34066	11307/21182
NCBM4	65261/75658	54262/57280	8/31	42120/32709	12134/24563
NCBS3	82706/84640	71188/61287	10/151	65493/25015	5688/36272
NCGR1	41951/72332	35359/53978	11/21	27956/34244	7403/19731
NCGR4	69287/85822	57476/62195	20/21	46548/41107	10919/21088
NCGR6	75101/82547	64603/64542	15/20	46797/40431	17798/24016

*Values correspond to 18SV1V2/18SV4.*

### *Symbiodinium* Diversity

Rarefaction curves for protists tended to level off for both marker regions, suggesting that most diversity was sequenced in each sample (**Supplementary Figure 2**).

**FIGURE 3:**
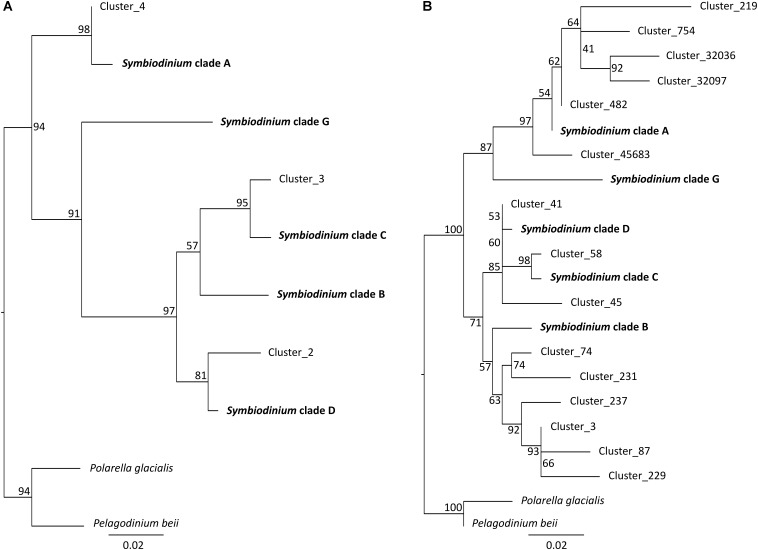
Phylogenetic analyses of environmental and reference sequences of *Symbiodinium*. **(A)** 18SV1V2 sequences. **(B)** 18SV4 sequences. Only representative sequences of environmental *Symbiodinium* were used for these trees. Representative sequences were identified using a clustering method, and a nucleotide identity of 95 and 97% for 18SV1V2 and 18SV4, respectively. The trees were rooted using two outgroups, *Polarella glacialis* and *Pelagodinium beii*. Numbers are bootstraps (%) reflecting clade support.

Not surprisingly, *Symbiodinium* accounted for most protist diversity within *P. damicornis* tissues for both primer sets (**Figure 2B**). In order to describe *Symbiodinium* diversity, we first estimated phylogenetic signals of both 18SV1V2 and 18SV4 compared to ITS2. ITS2 is the most common marker used to study *Symbiodinium* diversity (LaJeunesse et al., 2010; Wicks et al., 2010; Silverstein et al., 2011; Putnam et al., 2012; Tonk et al., 2013), since it has high polymorphism. The strength of correlation between the three markers was estimated using Mantel tests (**Table 4**). The three phylogenetic trees were significantly congruent (**Supplementary Figure 3**) (*p* = 0.003), but congruency with ITS2 was higher for 18SV1V2 (*r* = 0.88) than for 18SV4 (*r* = 0.72) (**Table 4**). We then annotated environmental *Symbiodinium* OTUs using a clustering approach and reference sequences of *Symbiodinium* clade A, B, C, D, and G (**Supplementary Table 4**). We also computed phylogenetic reconstruction with *P. glacialis* and *P. beii* as outgroups (**Figure 3**). While *Symbiodinium* clades A, C and D were common for both marker genes, strains from clade G were not identified with 18SV1V2 nor with 18SV4. Moreover, *Symbiodinium* clade B was found with 18SV4 but not with 18SV1V2, and we were not able to assign all environmental *Symbiodinium* to a known clade for 18SV4 in comparison to 18SV1V2 (**Supplementary Table 4**).

**Table 4:** Phylogenetic congruences between ITS2, 18SV1V2, and 18SV4 markers for *Symbiodinium*.

Marker 1	Marker 2	Correlation coefficient (r)	*p*-value
ITS2	18SV1V2	0.88	0.003
ITS2	18SV4	0.72	0.003
18SV1V2	18SV4	0.83	0.003

*Values correspond to correlation coefficients between patristic distances obtained using reference sequences of Symbiodinium and the Mantel test.*

### Diversity of the Other Dominant Genera

**FIGURE 4:**
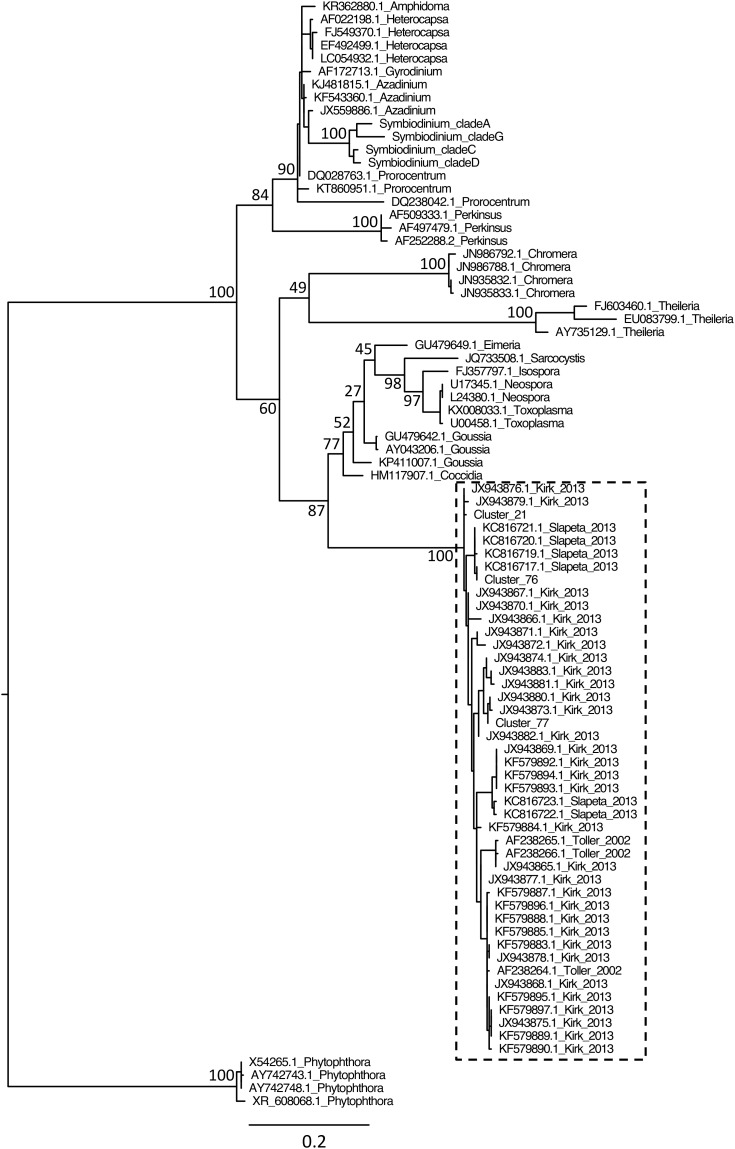
Phylogenetic analysis of coral symbionts related to the coccidian sequences of this study. Because we found more coral symbionts with BLASTn using 18SV4 than 18SV1V2, we only included coccidian OTUs of 18SV4 from this study (Cluster_21, Cluster_76, and Cluster_77) within the multiple alignment. Reference sequences of marine Apicomplexa and outgroups were selected according to a previous study (Schrével et al., 2016). The tree was rooted using *Phytophthora* strains as outgroups. Accession numbers and genus are indicated for each sequence (except for *Symbiodinium*, see **Figure 3** and **Supplementary Table 2**). Numbers are bootstraps (%) of major nodes reflecting clade support. The dashed box indicates coccidian OTUs and known sequences of coccidian symbionts associated to corals and their corresponding references (Toller et al., 2002; Kirk et al., 2013; Šlapeta and Linares, 2013).

Although protist proportion was lower for 18SV1V2 (**Figure 2A**), the proportion of protists other than *Symbiodinium* was lower for 18SV4 (0.9% compared to 2.9% for 18SV1V2) (**Figure 2B**). The *Symbiodinium* genus was removed from the dataset to study the other protist genera of *P. damicornis* (**Figure 2C** and **Supplementary Table 5**). *Licnophora*, unidentified coccidians and Dino-Group I-Clade 1 (Syndiniales) were the main taxa found in *P. damicornis* samples among the 17 genera found with both primer sets. Among them, *Licnophora* represented a high fraction for 18SV1V2 and 18SV4, whereas coccidians showed different proportions between these markers. In particular, 18SV1V2 showed a more even protist diversity at the genus level than 18SV4 (0.05 > 0.03, Pielou’s measure of evenness). A BLASTn search against NCBI nucleotide collection suggested that for both markers, *Licnophora* sequences were related to *Licnophora* strains, and that Dino-Group I-Clade 1 (Syndiniales) were similar to uncultured eukaryotes (**Table 5**). Interestingly, coccidian sequences were similar to protists already described in healthy coral colonies of *Agaricia agaricita*, *A. tenuifolia*, *Favia fragum*, *Montastraea annularis*, *M. faveolata*, *Mycetophyllia ferox*, *Porites astreoides*, and *Siderastrea siderea* (Toller et al., 2002; Kirk et al., 2013; Šlapeta and Linares, 2013). As a consequence, we computed a phylogenetic reconstruction of coral-associated coccidians with other Apicomplexa genera to describe their diversity. We found that all coral-associated coccidians formed a robust monophyletic clade (**Figure 4**). In addition, a phylogenetic tree using the longest available sequences of these symbionts highlighted their relationships with other marine Apicomplexa (**Supplementary Figure 4**), and confirmed that they corresponded to coccidians (Schrével et al., 2016).

**Table 5:** BLASTn search of coccidians, *Licnophora*, and Dino-Group I-Clade 1 (Syndiniales) against NCBI.

Marker region	Genus	Description	Identity (%)	Coverage (%)	*E*-value	Accession number
18SV1V2	Unidentified coccidia	Unidentified symbiont Type N clone N:0–1	96	100	1e-135	AF238264.1
	*Licnophora*	*Licnophora macfarlandi*	98	92	1e-128	AF527758.1
	Dino-Group I-Clade 1	Uncultured eukaryote clone SGYP555	100	100	6e-152	KJ763756.1
18SV4	Unidentified coccidia	Coral symbiont from *Montastraea faveolata* haplotype 12	99	100	0.0	JX943876.1
	*Licnophora*	*Licnophora macfarlandi*	96	100	2e-165	AF527758.1
	Dino-Group I-Clade 1	Uncultured eukaryote clone ST5900.009	100	100	0.0	KF129971.1

### Distribution of *P. damicornis*-Associated Protists

Because 18SV4 had (i) a low number of sequences related to protists other than *Symbiodinium*, (ii) low evenness for protist genera, and (iii) low phylogenetic signals (low congruency with ITS2 tree and low efficiency of annotations with reference *Symbiodinium* clades), we used 18SV1V2 amplicons to study protist distribution within the samples from Djibouti and New Caledonia.

**FIGURE 5:**
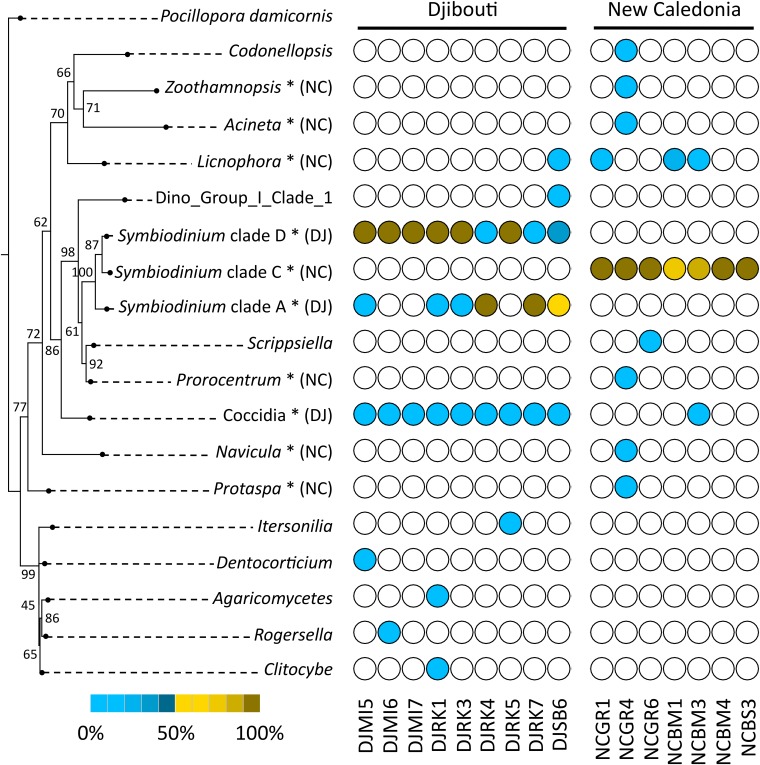
Phylogenetic diversity and distribution of *P. damicornis*-associated protists using 18SV1V2 marker region. The tree was rooted using *P. damicornis* as outgroup. Numbers are bootstraps (%) reflecting clade support. White circles indicate absence of taxa in samples. Brown circles indicate taxa frequency above 0.5. Blue circles indicate taxa frequency below 0.5. The gradient from light to dark colors indicates low to high frequencies of protists in each sample. ^∗^indicates significant taxa associated to Djibouti (DJ) or New Caledonia (NC) based on Fisher’s exact test.

A phylogenetic reconstruction of identified *Symbiodinium* clades and protist genera was carried out using maximum likelihood, and corresponding frequencies in samples were plotted in front of taxa (**Figure 5**). Two colors were used for frequencies to discriminate the high proportions of *Symbiodinium*, and lower values of other protists. Most protist genera were found in only one sample (e.g., *Codonellopsis*, *Zoothamnopsis*, *Acineta*, etc.). However, the different *Symbiodinium* clades, *Licnophora* and coccidians were present in several *P. damicornis* colonies. In particular, we observed different distribution between Djibouti and New Caledonia.

In order to statistically test differences between both geographic regions, we computed Fisher’s exact test for each protist genus and *Symbiodinium* clade (**Figure 5**). We found that coccidians and *Symbiodinium* clade D and A were linked to Djibouti, whereas *Licnophora* and *Symbiodinium* clade C were mostly associated with New Caledonia.

## Discussion

### Efficiency of Blocking Primers

First, a very high specificity was obtained *in silico* for both blocking primers. In accordance with sequence entropy values (**Supplementary Figure 1**), all Scleractinia from the Silva database were expected to be blocked for 18SV1V2, and we found that ∼94% of them were targeted by the blocking primers of 18SV4. Although both blocking primers matched with *Pocillopora* sequences, we observed various efficiencies for the different samples from Djibouti and New Caledonia. On average *Pocillopora* still represented 70% (from 30 to 92%) and 39% (from 7 to 66%) of sequences for 18SV1V2 and 18SV4, respectively. Such variations were also described for artificial rDNA mixtures of algae and krill (between 26 and 42% of krill sequences were not blocked by blocking primers) (Vestheim and Jarman, 2008), and for gut content of fish (between 14 and 45% of sequences were not blocked by blocking primers) (Leray et al., 2013). These variations might be related to (i) the ratio between host and total sequences (Vestheim and Jarman, 2008), (ii) the ratio between blocking primers and targeted primer set concentrations (Vestheim and Jarman, 2008), and (iii) the complexity of samples for sequence composition, *i.e.*, taxa diversity.

For environmental samples, the description of protist diversity might be improved by increasing the sequencing depth. In this exploratory study, we limited the number of sequences to an average of 60,000 per sample before the cleaning steps. One might also design blocking primers for *Symbiodinium* to increase the proportion of others protists. However, such an approach might fail if blocking primers for *Symbiodinium* and Scleractinia form primer dimers, and further if blocking primers for *Symbiodinium* target other closely related Suessiales or even alveolates. Indeed, other alveolates were previously identified in coral tissues (Moore et al., 2008).

### 18SV1V2 Is a More Suitable Marker Than 18SV4 to Explore Protist Diversity Within Corals

Protist diversity was mostly described using 18S rRNA and ITS2 markers in marine environments. While the 18S rRNA gene was effective to study the diversity of a wide phylogenetic range of taxa within a sample (Viprey et al., 2008; Bik et al., 2012; de Vargas et al., 2015; Tragin et al., 2016), ITS were more appropriate for closely related taxa (Arif et al., 2014; Su et al., 2017). Because ITS polymorphism is high, it offers a higher resolution than the 18S rRNA gene.

To date, ITS2 has been one of the most common markers used to describe *Symbiodinium* diversity (LaJeunesse et al., 2010; Wicks et al., 2010; Silverstein et al., 2011; Putnam et al., 2012; Tonk et al., 2013), because it provides enough resolution to describe *Symbiodinium* diversity within clades (LaJeunesse, 2002; Thornhill et al., 2017). In this study, we used two 18S rRNA markers to describe phylogenetically distant taxa, but the comparison of phylogenetic signals for *Symbiodinium* showed that 18SV1V2 was more congruent with ITS2 than 18SV4. This difference might explain why we easily annotated all environmental *Symbiodinium* for 18SV1V2 compared to 18SV4. In addition, the diversity of protist genera was more even for 18SV1V2 than for 18SV4, as *Symbiodinium* and *Licnophora* represented a lower proportion of protists using 18SV1V2.

Overall, in comparison to 18SV4, blocking primers and the primer set for 18SV1V2 showed a better phylogenetic signal for *Symbiodinium*, and a more even representation of protist diversity. Based on our findings, we recommend the use of 18SV1V2 to study protists associated with coral colonies.

### Different Distributions Between *Symbiodinium* Clade C and A/D

Because of the advantages of 18SV1V2 and because we obtained sequences for protists other than *Symbiodinium*, we focused our analyses on this marker to study the distribution of protist genera and *Symbiodinium* clades within the different samples.

In order to describe *Symbiodinium* diversity, we looked for reference sequences of the different clades that matched with 18SV1V2 and 18SV4. However, since ITS2 was the most common marker used so far, only representative sequences of clades A, B, C, D, and G were found. Unfortunately, although clade F was sometimes identified in Scleractinia (LaJeunesse, 2001; Rodriguez-Lanetty et al., 2003; Pochon et al., 2006), we were not able to use this clade to annotate environmental sequences. Clade G was described in other Anthozoa (Van Oppen et al., 2005; Bo et al., 2011), but not in scleractinian corals so far, thus it was not surprising that sequences of this clade were absent from our dataset. In contrast, clades A, C, D were the most common. In particular, clade C was dominant in New Caledonia, whereas clades D and A were mainly found in Djibouti. This result was similar to the analysis of the same samples using ITS2 (Brener-Raffalli et al., 2018). Thus, 18SV1V2 not only had a similar phylogenetic signal to ITS2, but also offered similar community composition for *Symbiodinium*.

### Coccidians and *Licnophora* Were the Two Other Main Taxa Within *P. damicornis*

Although eukaryotic microborers were common in coral colonies (Tribollet, 2008b; Pica et al., 2016), we did not find any of them in our samples. However, even though boring microflora inhabited live and dead corals, they were more abundant in the latter ones (Le Campion-Alsumard et al., 1995; Tribollet and Payri, 2001; Tribollet, 2008a). Moreover, in this study we did not crush coral skeleton (*i.e.*, where microborers inhabited), but instead, we extracted DNA from coral tissue. Similar to previous studies, we identified many Stramenopiles (Kramarsky-Winter et al., 2006; Harel et al., 2008; Siboni et al., 2010), and in particular different Bacillariophyta. Among them, the genus *Navicula* was present in one sample and was already isolated from the soft coral *Dendronephthya* (Hutagalung et al., 2014). We also found many fungi from the family Agaricomycetes (class Basidiomycota). Despite being a terrestrial mushroom-forming fungi (Hibbett, 2007), this family was already identified in many marine samples, from deep-sea sediments to oxygen-deficient environments, as well as within *Acropora hyacinthus* coral colonies (Amend et al., 2012). Many studies highlighted the presence of fungi in coral tissues: they were very diverse, and might be parasites, commensalists, and possibly mutualists that participated in nitrogen recycling (Wegley et al., 2007; Amend et al., 2012).

Furthermore, our samples contained many alveolates from divisions Dinophyta, Apicomplexa and Ciliophora. In particular, *Licnophora* (ciliates) and unidentified coccidia genera were the most common genera after *Symbiodinium* in *P. damicornis* colonies. Both ciliates and coccidians were already observed in coral samples using low-throughput methods, such as microscopy and culture, and they were mainly associated with coral diseases (Upton and Peters, 1986; Sweet and Bythell, 2012; Sweet et al., 2013; Sweet and Séré, 2016). However, the presence of *Licnophora* together with disease were possibly indirect, *i.e.*, resulting from a microbiota dysbiosis, since they are known to feed others protozoa (Sweet and Séré, 2016). Moreover, coccidians were also found within healthy coral colonies of *A. agaricita*, *A. tenuifolia*, *F. fragum*, *M. annularis*, *M. faveolata*, *M. ferox*, *P. astreoides*, and *S. siderea* (Toller et al., 2002; Kirk et al., 2013; Šlapeta and Linares, 2013). In this study, corals did not show any outward signs of pathology, suggesting that these genera might be commensalists or mutualists. Interestingly, coccidian sequences of this study were very similar to the other coral-associated coccidians, and these sequences formed a robust monophyletic clade within Apicomplexa. This observation suggested that a speciation event of coccidians was linked to interactions with corals. Future studies should test the role of coccidians in coral holobionts. For example, it would be interesting to know whether these coccidians have retained a relict or a functional plastid like the coral-associated chromerids (Janouškovec et al., 2012).

Finally, *Licnophora* and coccidians had different distributions within our samples from Djibouti and New Caledonia. Similarly to *Symbiodinium* clades, geographic locations, *Pocillopora* clades and thermal regimes might influence their distribution. However, because of our sampling strategy, it was not possible to identify the factors responsible for this pattern.

To conclude, we designed two blocking primers to characterize protist diversity using high-throughput amplicon sequencing for the first time within coral colonies. We were able to characterize the diversity of *Symbiodinium* and of other less known genera associated with *P. damicornis sensu lato*. Among them, *Licnophora* and unidentified coccidia genera were common in coral samples from Djibouti and New Caledonia. In particular, coccidian sequences were phylogenetically related to coccidians described in other scleractinian coral species. Furthermore, different distributions were highlighted between *Licnophora* and coccidians, and between *Symbiodinium* clades C and A/D. Because the dataset was limited to two geographic regions, we did not know the respective influence of geography, *P. damicornis* clades or thermal regimes on protist assemblages. Moreover, we could not confirm that *Licnophora* and coccidians were part of the coral holobiont, and not simply just a part of the larger environmental microbial community. Notably, future studies should decipher if they serve a specific function within the holobiont. However, we believe that these blocking primers are promising tools to bring new knowledge and understanding of the diversity and distribution of protists within *P. damicornis* colonies, as well as for other species of corals, as they were designed to target most Scleractinia.

## Author Contributions

CC, J-ME, and ET conceived the project. SB and PL designed the experimental protocol to test blocking primers. JV-D and MA were involved in the collection of samples and data acquisition. CC, LG, and ET performed the analyses. CC drafted the manuscript. All authors contributed to critical revisions and approved the final manuscript.

## Conflict of Interest Statement

The authors declare that the research was conducted in the absence of any commercial or financial relationships that could be construed as a potential conflict of interest.
