# Supplementary figures and images for: Protists Within Corals: The Hidden Diversity
Source: Front Microbiol. 2018 Aug 31;9:2043. doi: 10.3389/fmicb.2018.02043 (PMC6127297; doi:10.3389/fmicb.2018.02043)

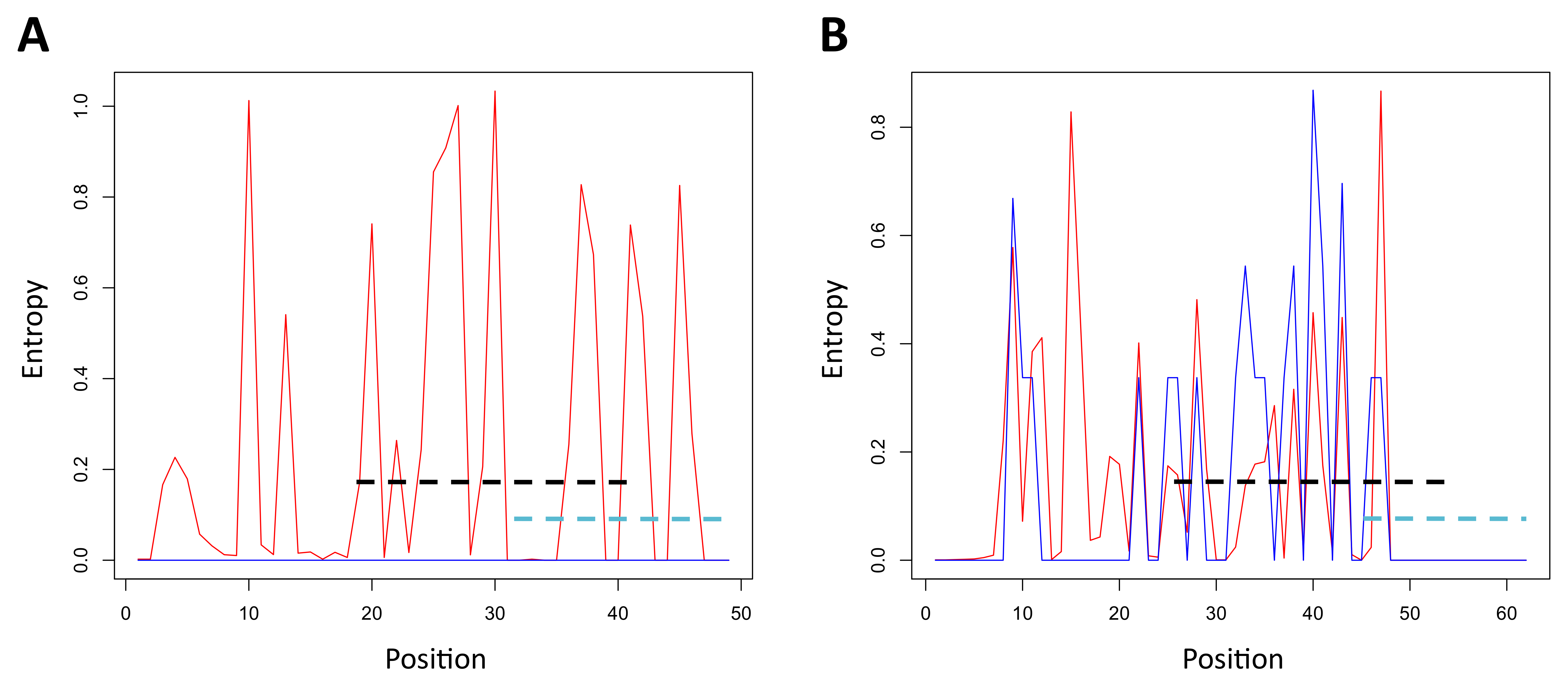

Supplement: Supplementary file 1 [file Image_1.TIFF]

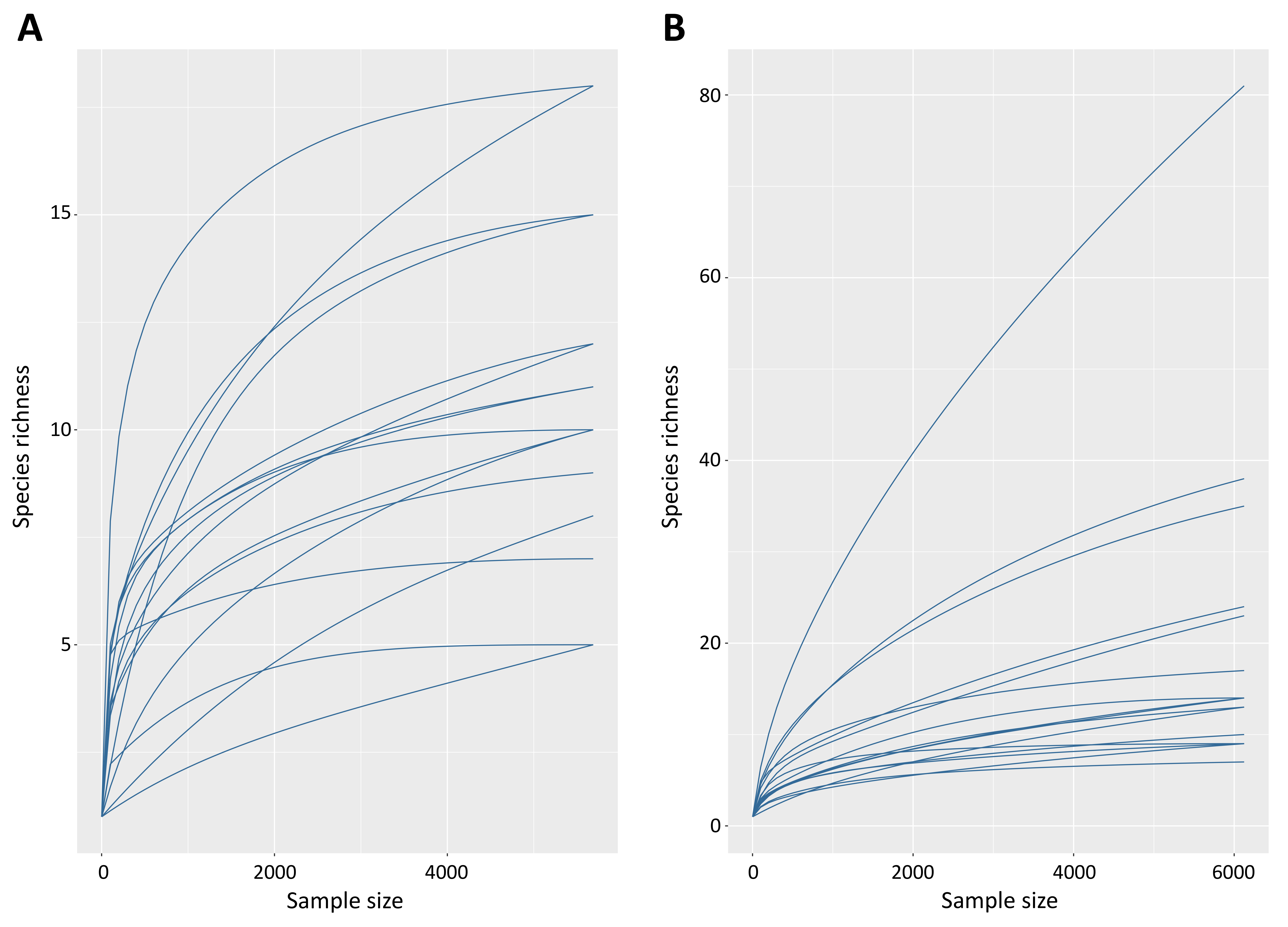

Supplement: Supplementary file 2 [file Image_2.TIFF]

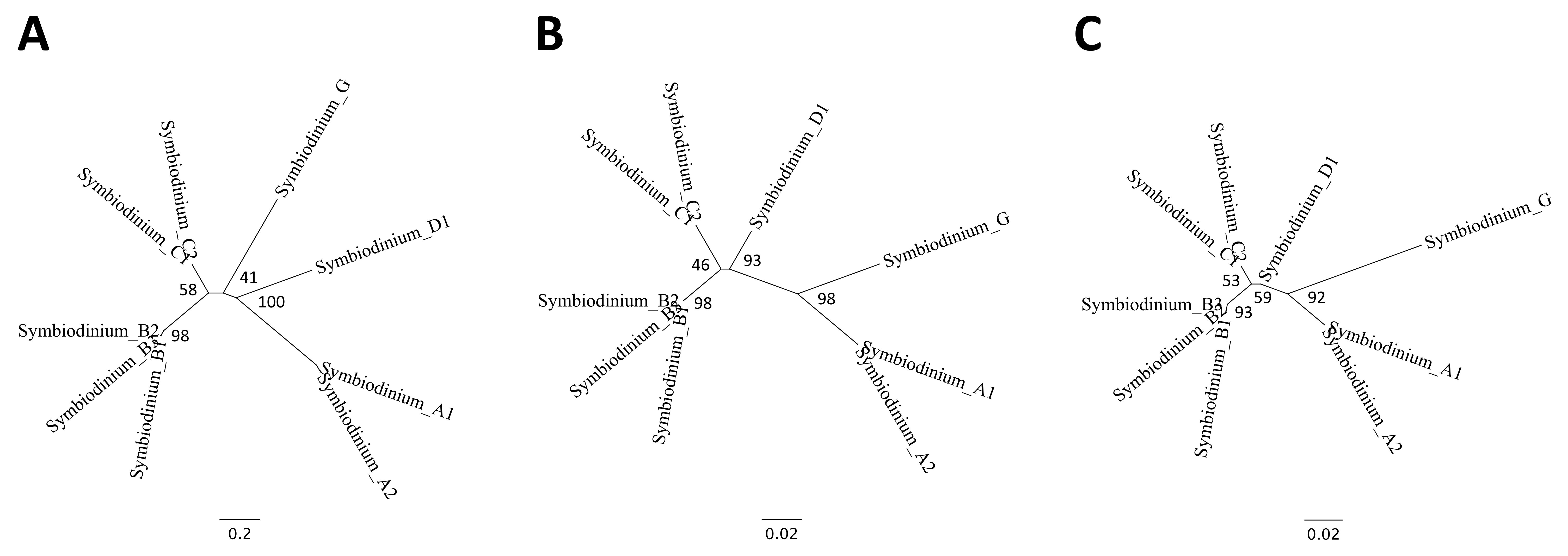

Supplement: Supplementary file 3 [file Image_3.TIFF]

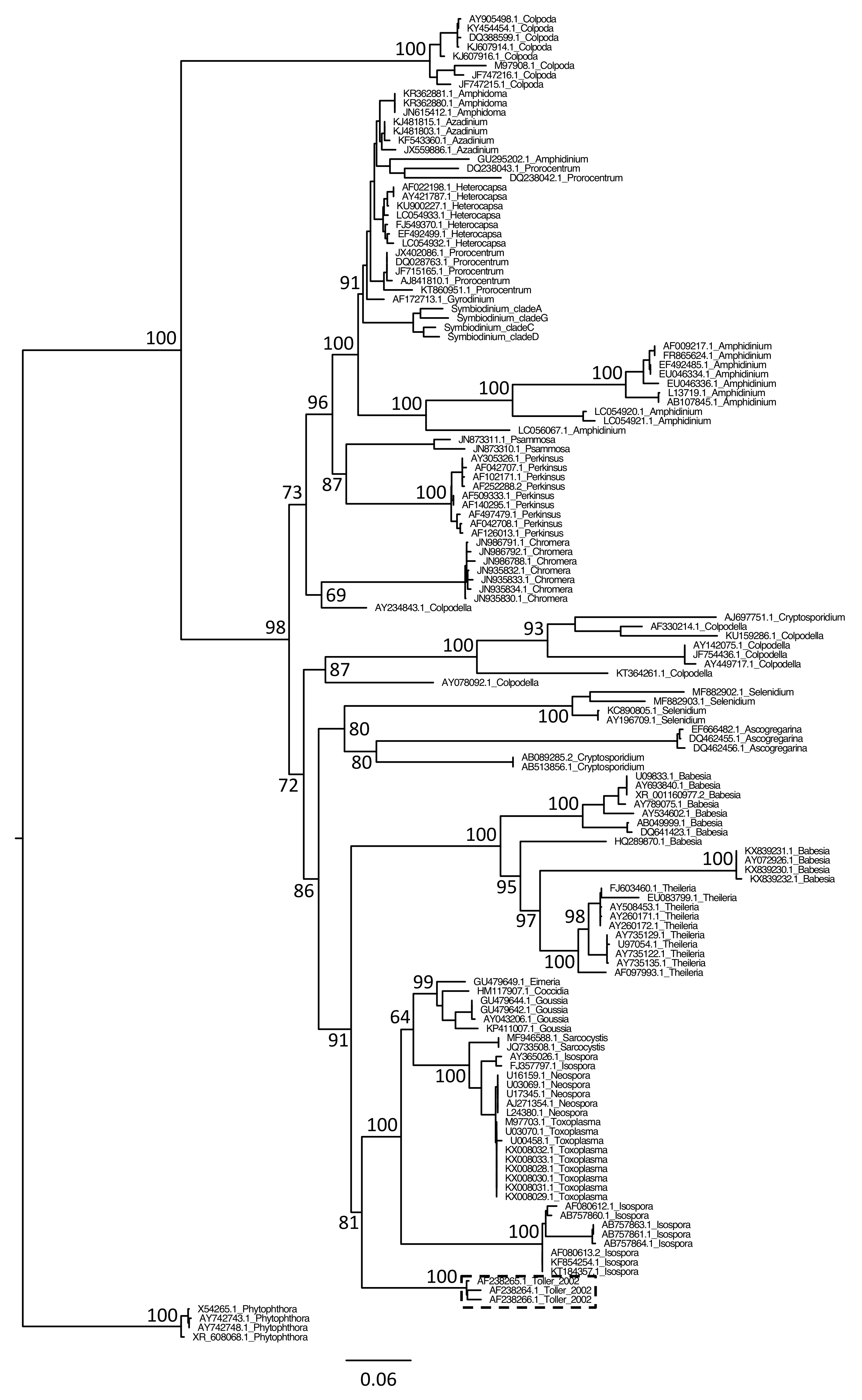

Supplement: Supplementary file 4 [file Image_4.TIFF]
